# Letter of Instructions to Medical Societies in Affiliation with the State Medical Association

**Published:** 1902-05

**Authors:** H. A. West

**Affiliations:** Secretary; Galveston, Texas


### Letter of Instructions to Medical Societies in Affiliation With the State Medical Association.

GALVESTON, TEXAS, March 27, 1902.

*Dr. W. B. Russ, Secretary West Texas Medical Association, San Antonio, Texas.*

MY DEAR DOCTOR: I beg to advise you that in accordance with the recommendations of the Committee on Organization of the

American Medical Association, the president of the State Medical Association of Texas, Dr. Taylor Hudson, appointed the following as a Committee on Revision of our constitution and by-laws, viz.: Drs. J. T. Wilson, Sherman; S. C. Red, Houston; J. E. Gilcreest, Gainesville; R. F. Miller, Sherman, and H. A. West, Galveston. This committee, or a part thereof, will present a report at our next annual session at Dallas May 6-9, embracing the fundamental provisions as recommended by the Committee on Organization, viz.: an agreement to federate ourselves in the American Medical Association and adopt, as far as possible, an organic law uniform with other States in regard to certain basic principles, as follows:

1. To divide our annual meeting into two branches, legislative and scientific; the legislative branch to be as small as compatible with representation from all societies in affiliation and to be composed of delegates elected by the county and district societies.

2. That membership in the county and district society shall constitute membership in the State society without further payment of dues, and that no one shall be admitted to membership except through affiliated societies.

3. That funds to meet expenses of the State Association shall be raised by a per capita assessment on the county and district societies.

4. That we will create a permanent committee and fund for the purpose of enforcing all medical laws in the State of Texas.

5. That we will co-operate with the American Medical Association and the other State societies in solving problems now before the profession relating to medical education, legislation, reciprocity, licensing, etc.

The report will further recommend that the initiation fee be dispensed with and that annual dues be reduced to $3.00, and as soon as possible still lower. Also that the proceedings of the State Association and of its affiliated societies, as far as may be practicable, shall be published in a monthly journal in lieu of the small volume of transactions.

The basis of representation for the legislative branch to be offered will be as follows: Each county society shall be entitled to one delegate. The district branches shall be represented upon the basis of one delegate to each one hundred members and fraction over thirty. It is suggested that in view of the probable adoption of the foregoing or similar revision that every county and district society in the State which proposes to accept the invitation of the State Medical Association to become one of its affiliated branches upon the above mentioned basis, proceed at once to elect its delegates that they may be present at Dallas and participate in the reorganization.

In this connection let me call your attention to the fact that admission hereafter to the American Medical Association will depend upon membership in the State societies and their affiliated branches. In the event that your society does not hold a meeting before May 6, the delegates may be appointed by the president. I beg to remind you that a condition precedent for the recognition of delegates is the payment of your per capita assessment of ten cents per member. This assessment if not already paid should be forwarded without delay to Dr. R. F. Miller, treasurer, Sherman, Texas. (See Constitution, Article VII, Sec. 1.)

Trusting your society will appreciate the necessity of prompt action in the matter, I have the honor to be,

Fraternally yours,

(Signed) H. A. West, Secretary.

P. S.—Please ascertain as soon as it is possible to do so, whether the society of which you are an officer will accept the invitation of the State Association to become one of its affiliated societies on the above mentioned basis or some immaterial modification of the same, and notify me the result. H. A. W.

